# Tonometry and Care of Tonometers

**DOI:** 10.5005/jp-journals-10008-1119

**Published:** 2012-10-16

**Authors:** Rajat Maheshwari, Nikhil S Choudhari, Manav Deep Singh

**Affiliations:** Consultant and Ophthalmologist, Department of Glaucoma, Maheshwari Eye Center Muzaffarnagar, Uttar Pradesh, India; Associate Ophthalmologist, Department of Glaucoma and Neuro-ophthalmology, VST Glaucoma Centre, LV Prasad Eye Institute, Hyderabad, Andhra Pradesh, India; Associate Professor, Department of Ophthalmology, Postgraduate Institute of Medical Education and Research, Chandigarh, India

**Keywords:** Tonometry, Tonometers, Intraocular pressure.

## Abstract

Intraocular pressure (IOP) remains the only modifiable risk factor in the management of glaucoma. Hence, IOP and its appropriate measurement deserve our ongoing interest. Over the years, not only has our understanding of glaucoma changed but also has changed our approach to the measurement of the IOP. This review is an attempt to elucidate the commonly techniques of tonometry, and critically evaluate each of them, in current glaucoma practice.

**How to cite this article:** Maheshwari R, Choudhari NS, Singh MD. Tonometry and Care of Tonometers. J Current Glau Prac 2012;6(3):124-130.

## INTRODUCTION

Definition of glaucoma has changed over the decades from a simple ocular pressure-related disease to a systemic disorder of multivariate etiology. However, intraocular pressure (IOP) remains the only modifiable risk factor in the management of glaucoma. Hence, IOP and its appropriate measurement deserve our ongoing interest. Over the years, not only has our understanding of glaucoma changed but also has changed our approach to the measurement of the IOP. Tonometry has been over 180 year’s long journey.^1^ In early 19th century, Sir William Bowman at British medical association addressed the critical role of digital estimation of ocular tension. In late 19th century, Donders designed the first instrument which displaced intraocular fluid by contact with the sclera. The first commonly used mechanical tonometer designed by H Schiotz in early 1900s soon became the new gold standard.

### Schiotz Indentation Tonometer

**Fig. 1:**
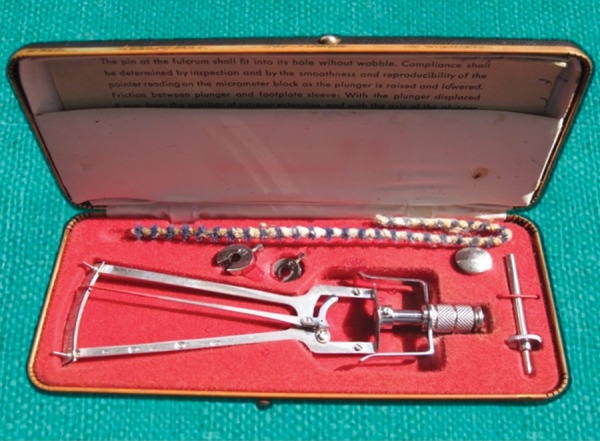
Schiotz tonometer

**Fig. 2:**
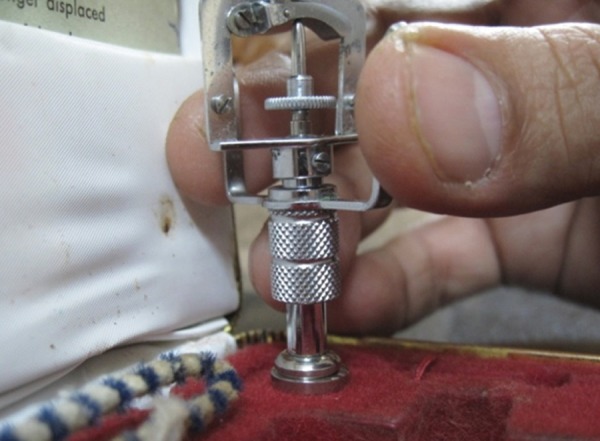
Checking Schiotz tonometer for zero error

Schiotz tonometer (Fig. 1) works on the basic concept of indentation tonometry. The body of the tonometer has a footplate, which rests on the cornea. A plunger moves freely (except for the effect of friction) within a shaft in the footplate (Fig. 2) and the degree to which it indents the cornea gives an estimate of the IOP. Although not considered a standard instrument in glaucoma care, it is still in use because it is reasonably reliable, simple, has no electronic component, is inexpensive to use and maintain, it is calibration is simple and because it can be used in restricted patients.

**Fig. 3:**
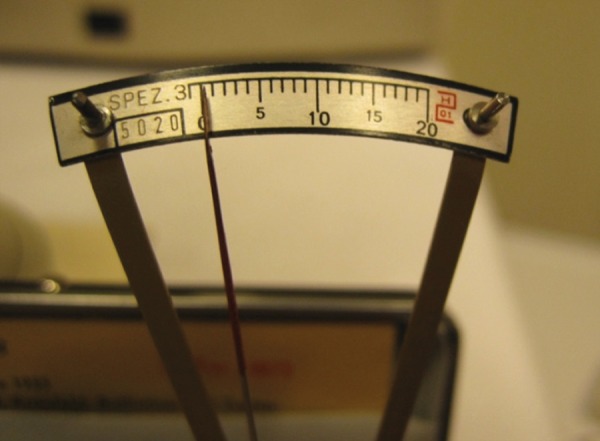
Checking the Schiotz tonometer on a dummy cornea before use

### Technique

With the patient in supine position and fixing on a target just overhead, the examiner separates the eyelids and gently rests the tonometer footplate on the anesthetized cornea in a position that allows free vertical movement of the plunger. When the tonometer is properly positioned, the examiner observes a fine movement of the indicator needle (Fig. 3) on the scale in response to the cardiac pulsations. The scale reading should be taken as the average between the extremes of these excursions. It is customary to start with the fixed 5.5 gm weight. However, if the scale reading is 4 or less, additional weight should be added to the plunger. A conversion table is then used to derive the IOP in mm Hg from the scale reading and plunger weight.

### Sources of Error with Schiotz Tonometry

The accuracy depends on the assumption that all eyes respond the same way to the external force of indentation, which is not the case. The following are some of the more common variables that introduce potential for error.

- Ocular rigidity–the instrument is grossly unreliable for IOP measurement following glaucoma or vitreoretinal surgery or open globe injuries due to low scleral rigidity in these conditions.
- Blood volume alteration.
- Corneal influences–steeper or thicker cornea leads to a falsely high IOP reading.^2^
- Moses effect.^3^

### Care of Schiotz Tonometer

**Fig. 4:**
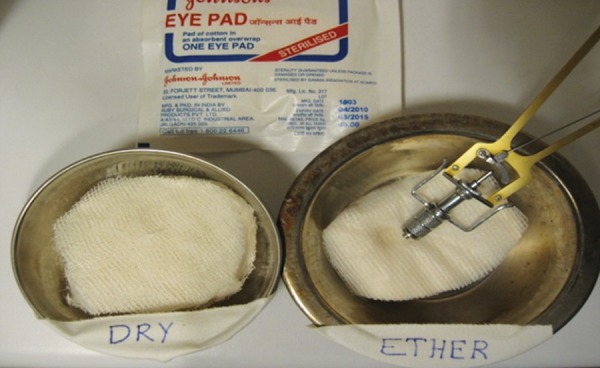
Cleaning of Schiotz tonometer

- Zero error–should be checked before the day starts
- Cleaning in between cases–ether or sodium hypochlorite can be used to disinfect the tonometer in between cases (Fig. 4).
- Cleaning of barrel–should be done daily to avoid plunger sticking to the barrel.
- Storage–should be done in dry, dust free environment with separable parts separated.

**Fig. 5:**
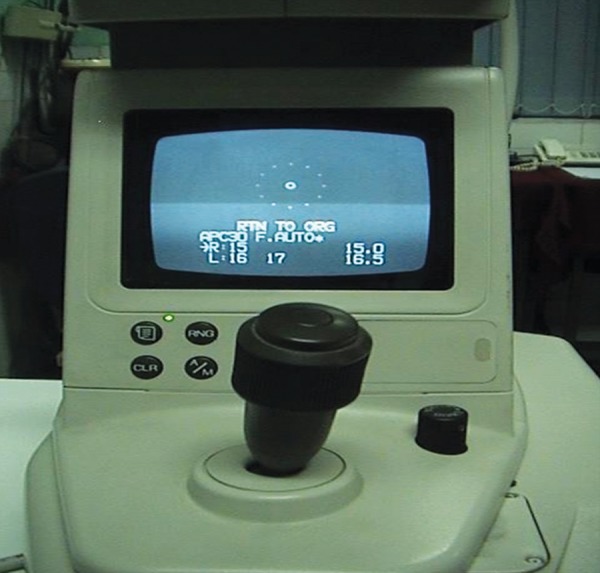
Noncontact tonometer

**Fig. 6:**
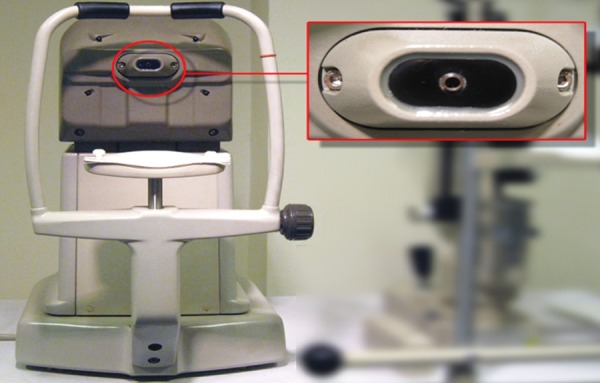
Noncontact tonometer sensor

### Noncontact Tonometer

The Noncontact tonometer (NCT; Fig. 5) was introduced by Grolman^4^ and has the unique advantage over other tonometers of not touching the eye, other than with a puff of air. A pneumatic system generates a puff of air which is directed against the cornea and a detector device estimates the IOP based on reflections from flattened cornea. In most studies, comparisons against Goldmann applanation tonometers indicate that the NCT is reliable within the normal IOP range, although the reliability is reduced in the higher pressure ranges and is limited by an abnormal cornea or poor fixation.^5-7^ Corneal thickness has a greater influence on NCT readings than on Goldmann tonometry.^8^

A significant advantage of the NCT is the elimination of potential hazards associated with all contact tonometers viz. (a) abrasion of cornea (b) reactions to topical anesthetics and (c) spread of infection. In addition, the instrument can be used reliably by paramedical personnel and has particular value in mass screening. However, a remote chance that infection could be spread by contamination of the front surface of the instrument with tear film at the time of air impact has been reported.^9^

Care of NCT–the plastic parts can be cleaned using a mild detergent. The sensor needs regular cleaning with soft tissue (Fig. 6).

### Goldmann Applanation Tonometer

Goldmann applanation tonometer (GAT) is the current standard against which all other tonometers are compared. Most tonometers, including GAT work based on the Imbert-Fick principle, which states that the pressure (P) inside a sphere is equal to the force (F) necessary to flatten the surface divided by the area (A) of flattening (P = F/A). However, the eye is neither a perfect sphere nor is infinitely thin (as presumed in law). Additionally, corneal rigidity resists the force and the capillary action of the tear film attracts the tonometer prism. GAT is designed such that these two opposing forces approximately cancel each other when the applanated area is of 3.06 mm diameter.

### How to use GAT?

Before undertaking Goldmann applanation tonometry certain precautions need to be taken viz.^10^

**Fig. 7:**
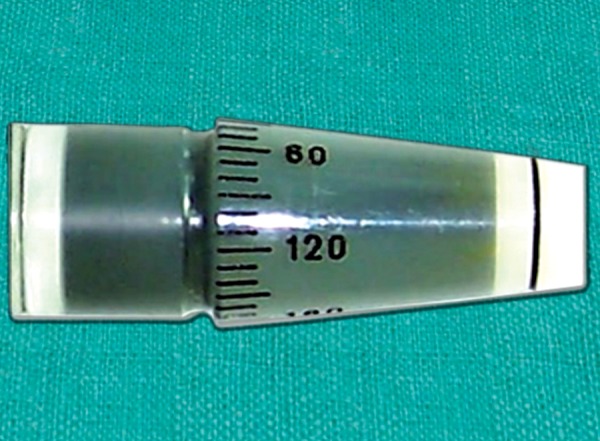
Goldmann Applanation tonometer prism

- Avoid tonometry in infected or injured eyes
- Clean prism before every use
- Disinfect prism before first use and when indicated
- Wait adequately for the cleaned surface to dry
- Replace prism every 2 to 3 years
- Unused prism can be kept indefinitely
- Verify prism for scratches/sharp edges and cracks because scratches can injure cornea and disinfectant can get into hollow of cracks and can cause chemical injury to cornea (Fig. 7).

**Fig. 8:**
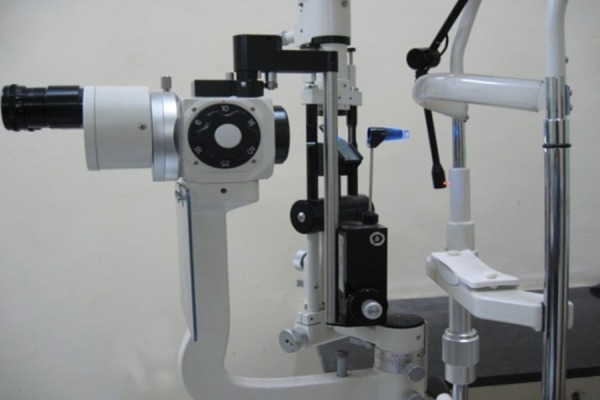
Goldmann Applanation tonometer attached to the slit-lamp

*Procedure*: Insert the prism into the tonometer holder, ensuring the 0° or 180° markings line up with the white line on the bracket. GAT is usually mounted permanently on the slit lamp; if not, mount the instrument on the slit lamp. Swing the tonometer round to locate it centrally in the measuring position. Prism and the tonometer should get fixed in notch.

**Figs 9A and B:**
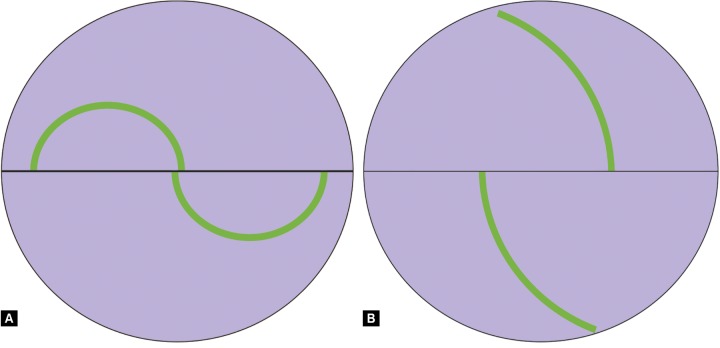
Alignment of mires

**Fig. 10:**
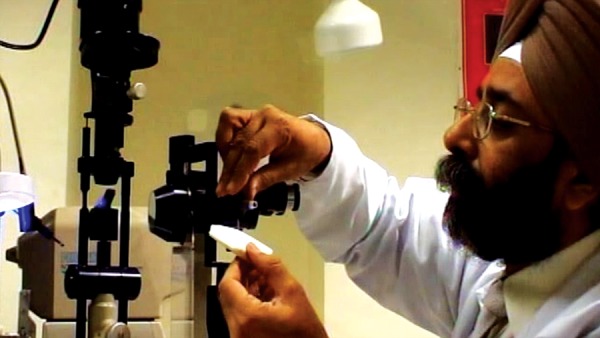
Goldmann Applanation tonometry (a video presentation of GAT can be seen at: http://www.youtube.com/watch?v=Zx0xslEv9q0)

- Increase the light source to maximum intensity with the cobalt blue filter on and the slit opened fully. It should illuminate the prism from the side at about 60° (Fig. 8).
- Explain the procedure to the patient. Instill a drop of anesthetic into the eyes.
- Positioning and cooperation of the patient are vital. Ensure the patient is comfortable with the chin on the chin rest and forehead firmly against the forehead bar. Ask the patient to look straight ahead with eyes wide open.
- Advance the slit lamp toward the eye with the joystick. When the tip of the prism is within a centimeter or so of the cornea, use the joystick to gently bring the tip into contact with cornea under direct vision. The limbus will light up when you have made contact.
- Look through the slit lamp eyepiece (only one eyepiece, usually the left, is lined up with the prism). You should see two semicircles of fluorescein shifted away from each other along the horizontal axis.
- Use the slit lamp joystick to position the semicircles at the center of the prism. Now adjust the dial to alter the force on the prism and thus alter the size and overlap of the semicircles. The end point is regular pulsation of two semicircular rings of equal size the inner edges of which just interlock (Fig. 9A). These pulsations represent cardiac cycles. The correct value is the midpoint of this variation. The IOP in mm Hg is the value on the dial multiplied by ten (Fig. 10).

### Common Sources of Error and Ways to avoid them

- Patient should not hold breath during tonometry or have tight collar around neck. This can lead to overestimation of IOP.
- Patients are often unable to keep the eyes open without blinking, in which case you must gently hold open the lids with one hand. It is important not to apply any pressure to the globe, as this would increase the measured IOP. To avoid this, hold the lids against the orbital rim.
- Ensure the tonometer tip does not touch eyelashes. They are not anesthetized and this will induce blinking.
- Large overlapping semicircles (Fig. 9B), which do not pulsate and do not change size when the measuring dial is turned, indicate over-applanation. Ensure the slit lamp is not moved too far forward.
- The width of the fluorescein semicircles should be about 1/10th the diameter of the ring. A thin ring indicates insufficient fluorescein. This will underestimate the pressure. Ask the patient to blink or instil more fluorescein and try again. If there is too much fluorescein in the tear film or the prism touches the upper lid, a thick ring will be seen, resulting in overestimation of the pressure. Dry the prism and repeat.
- Inaccurate IOP readings can result from the presence of abnormal corneal thickness or corneal pathology. One may correct the measured IOP level for the pachymetry value of the patient using an appropriate correction formula.
- Repeating the measurement allows you to take an average to improve the accuracy.^4^

**Figs 11A and B:**
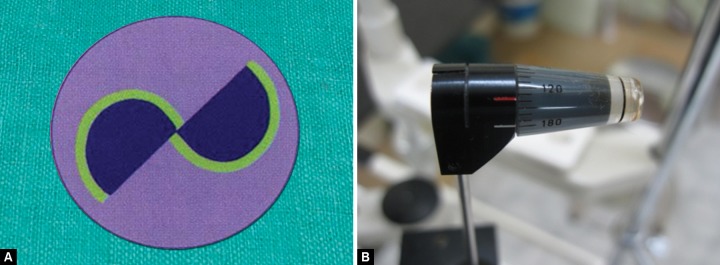
Tonometry in patients with marked astigmatism

### Patients with Marked Astigmatism

In patients with astigmatism of greater than three-dimension (3D), the applanated area will be elliptical, not circular. This error can be avoided by applanation at 43° to the axis of minus cylinder. This is done by lining up the angle of minus cylinder on the prism with the red mark on the prism holder (Figs 11A and B).

### Calibration Error of the Goldmann Tonometer

Calibration error of GAT is not uncommon.^11^ One can check the instrument for calibration error.^10^ This is done at dial position 0, 2 and 6 (0, 20 and 60 mm Hg equivalents).

**Fig. 12:**
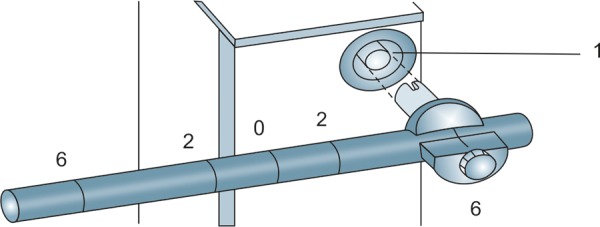
Calibration of Goldmann Applanation tonometer

- Insert the prism in the holder and place the tonometer on the slit lamp.
- At setting 0, if the dial position is moved to –0.05 the feeler arm should fall toward the examiner; if the drum is moved to position +0.05 the arm should fall toward the patient.
- To check settings 2 and 6, the calibration error check weight bar provided by the manufacture is used.
- The calibration error check weight bar has 5 markings on it. The central marking corresponds to level 0. Two on either side of it represent level 2 and the two outermost markings represent level 6. These markings correspond to 0, 20 and 60 mm Hg of IOP respectively.
- The calibration error check weight bar and holder are fitted into the slot provided on the side of the applanation tonometer (Fig. 12). After setting the mark on the weight bar corresponding to position 2 or 6 on the index mark of the weight holder, the measuring drum is rotated forward. The reading at which the feeler arm with the prism in place moves forward freely is recorded. The difference of this reading from the respective test position is the positive error at that level of testing. Similarly, on rotating the revolving knob in the reverse direction, the reading at which the feeler arm moves backward is noted. The difference between the latter and the testing position is the negative error at that level of testing.
- The manufacturers of Haag-Streit GAT accept calibration errors within ±0.5 mm Hg at all levels of testing (0, 20 and 60 mm Hg).^10^ On the other hand, the South East Asia Glaucoma Interest Group (SEAGIG) guideline is less stringent and recommends that the acceptable range of calibration error should progressively widen at the higher levels of error testing. By this guideline, the acceptable error could be within ±2 mm Hg at 0 mm Hg, ±3 mm Hg at 20 mm Hg and ±4 mm Hg at 60 mm Hg testing levels.^12^
- If a GAT has unacceptable calibration error, the instrument should be sent to the manufacturer for rectification of the error. One should avoid estimating true IOP from a faulty GAT by applying a correction factor as the calibration error of GAT has high variability.^13^

**Fig. 13:**
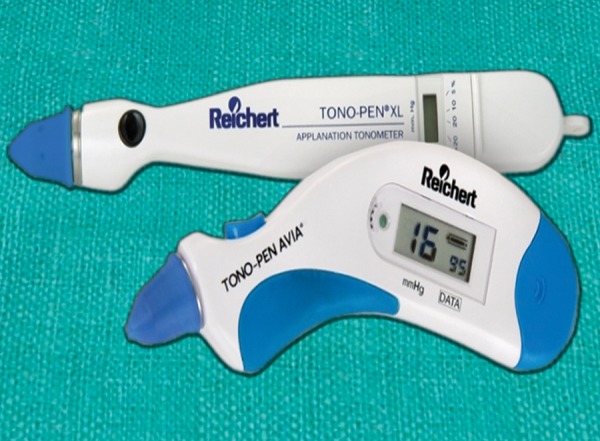
Tono-Pen AVIA

### How to disinfect GAT Prism?

- Separate the tonometer probe from the prism holder/ feeler arm.
- Rinse the prism in running cold water for 30 to 60 seconds and wipe clean.
- Place in a disinfectant solution of either 10% sodium hypochlorite or 3% hydrogen peroxide for 10 minutes.^10^
- Remove the probe from the solution and place in a clean container. Rinse with cold running water for no more than an hour and no less than 10 minutes.
- Dry the probe. Use a soft, clean tissue and wipe only once in a single direction starting at the probe tip.
- One may simply wipe the tip of the prism with 70% isopropyl alcohol or 2% bacillocid swab. Allow sufficient time for the tip to dry before subsequent use. This method though time saving, may not kill all the pathogens.^14^

### Perkins Tonometer

The Perkins tonometer works on the same principle as the Goldmann but is portable and does not require a slit lamp, allowing measurement of the supine patient. It is often more difficult to get accurate readings.

### Tono-Pen

The Tono-Pen is an applanation tonometer based on the earlier Mackay-Marg design. Its tip incorporates a stainless steel transducer which uses microstrain gauge technology to convert pressure into an electrical signal (Fig. 13). The signal is analyzed and displayed as the final IOP on a quartz crystal screen as an average of 4 readings, along with a statistical coefficient. In the normal population, Tono-Pen has been found to be consistent and accurate comparative to GAT in the mid-IOP range. Results are less accurate in the 4 to 10 mm Hg and 21 to 30 mm Hg intervals, and considered unreliable over 30 mm Hg where it can significantly underestimate the pressure. Tono-Pen is useful for children, supine patients and when there is a gross corneal pathology.

### How to use the Tono-Pen?

- Ensure that there is a new rubber cover over the tip. Instil an anesthetic drop into the eye. Turn on the Tono-Pen by holding down the button. It will require calibrating on start-up. This is done by holding Tono-Pen tip down, then when it displays ‘UP’ on the display, hold it tip pointing upward. However, the recent models do not require calibration before every use.
- Repeatedly tap the Tono-Pen gently on the central cornea; it beeps with each reading then gives a longer beep when it has enough results. An average value will be displayed on the screen along with a 5% (best) to >20% confidence value.
- Do not leave the Tono-Pen without a rubber cover on the tip in its box as this protects the tip from dust.

### Dynamic Contour Tonometer

**Fig. 14:**
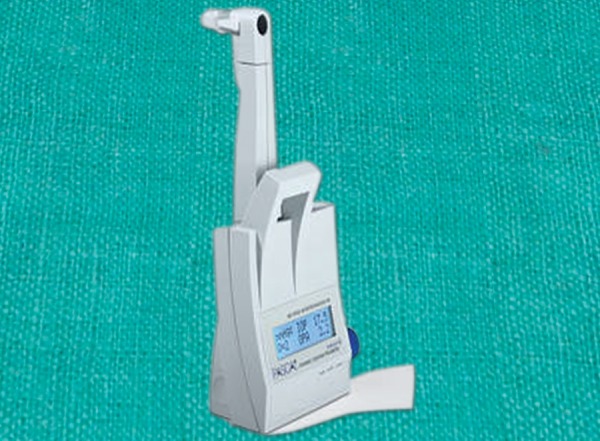
Pascal dynamic contour tonometer

The Pascal dynamic contour tonometer (DCT) is a contact tonometer similar in appearance to GAT and is attached to a slit-lamp by a metal footplate (Fig. 14). It has an integrated solid-state piezoelectric pressure sensor which permits a direct IOP measurement free of observer errors inherent in GAT. The contour of the contact surface matches the cornea during tonometry and thereby accommodates physical variations in cornea.

An auditory signal informs the examiner of ‘good’ contact with the anesthetized cornea during tonometry. An average contact time of 5 seconds is usually required. During this time, up to 100 measurements are taken per second, and the average minimum reading is displayed on the digital LCD screen, with a Q value indicating reliability. The tips are available with disposable covers to eliminate the risk of transferring microorganisms.

Another good feature of the instrument is its ability to self-calibrate. A measure of ocular pulse amplitude (OPA) is also displayed with each IOP reading, enabling quantification of IOP fluctuations with cardiac pulse. OPA provides an estimation of ocular blood flow to the optic nerve head.

Pascal DCT has been shown to correlate well with manometric IOP measurements.^15^ Several clinical publications support the hypothesis that DCT measurements are not significantly affected by changes in CCT, keratometry or corneal biomechanics. Thus, it appears to be promising in assessment of postrefractive surgery cases (but not post keratoplasty). However, its use does not do away with the need to measure CCT because although more accurate than GAT in thin corneas, it overestimates IOP in high CCT.

### Tonometry in Supine Position

An average rise of 2 to 3 mm Hg in IOP, when the body position is changed from sitting to supine has been known. The elevation of IOP occurs due to the obstruction of aqueous outflow by an increase in episcleral venous pressure as the episcleral venous pressure depends on the position of the ocular structure relative to that of the heart. A recent study showed more rise in IOP in patients with primary open angle glaucoma (POAG) compared to normal subjects upon moving them from a sitting to supine position.^16^ Moreover, the greatest difference in IOP between the sitting and supine positions was observed in the worse eye of patients with POAG.^16^ Of the described tonometers, only Perkin’s tonometer, Tonopen and Schiotz Tonometer can be used to obtain IOP in supine position.

### Diurnal and 24-Hour IOP Monitoring

While elevated IOP is the most recognized risk factor for glaucoma, diurnal IOP fluctuation has also been shown to be a risk factor in the development and progression of glaucoma.^17^ Moreover, nocturnal IOP has been shown to be higher than diurnal IOP in patients both with and without glaucoma.^18,19^ In clinical practice, we primarily rely on measurements of IOP obtained during office hours and in the sitting posture, to make decisions in glaucoma. The office IOP measurements may not provide total information on IOP fluctuation and one should exercise caution when extrapolating data and making treatment decisions based on office IOP readings.

This is evident from the study by Barkana et al.^20^ They found peak IOP outside of office hours in 22 (69%) patients. Also, the mean peak 24-hour IOP was higher than the peak office IOP. Similarly, the mean IOP fluctuation during 24-hour monitoring was significantly higher than IOP fluctuation acquired during office hours. Moreover, in 40 (62%) eyes, the peak 24-hour IOP was higher than any IOP recorded during previous office visits. Therefore, examining glaucoma patients periodically at different times of day and different days of the year may not suffice, at least in some patients.

Another study also found wider IOP fluctuation outside office hours than with in-office measurements.^21^ The mean peak 24-hour IOP averaged 4.9 mm Hg higher than mean peak IOP measured in office. Twenty-four hours IOP monitoring resulted in a change of clinical management in 23 (79.3%) patients.

Nevertheless, obtaining 24-hour IOP is logistically difficult. Therefore, it is considered in selected situations. The prominent among them are glaucoma progression despite good office IOP control and reasonable patient compliance to the medications, diagnosing normal tension glaucoma, and patients with advanced glaucoma or monocular patients who have normal office IOP measurements on treatment to rule out IOP peaks outside office hours. One may choose situations to perform day-diurnal IOP recordings which are logistically easier. A recent contact lens based IOP sensor that records the change in corneal curvature and thereby the corresponding IOP for 24 hours shows promise to easier collection of continuous 24 hours IOP recording.^22^ Moreover, this device promises to avoid possible artefacts associated with nocturnal arousal.^22^
